# Orthodontic Treatment of a Patient with Dentin Dysplasia Type I and Bilateral Maxillary Canine Impaction: Case Presentation and a Family-Based Genetic Analysis

**DOI:** 10.3390/children8060519

**Published:** 2021-06-18

**Authors:** Alexandros Papagiannis, Galinos Fanourakis, Anastasia Mitsea, Kety Karayianni, Heleni Vastardis, Iosif Sifakakis

**Affiliations:** 1 Department of Orthodontics, School of Dentistry, National and Kapodistrian University of Athens, 2 Thivon Str, 11527 Athens, Greece; vastard@dent.uoa.gr (H.V.); isifak@dent.uoa.gr (I.S.); 2 Department of Oral Biology, School of Dentistry, National and Kapodistrian University of Athens, 2 Thivon Str, 11527 Athens, Greece; gal.fanourakis@gmail.com; 3 Department of Oral Diagnosis and Radiology, School of Dentistry, National and Kapodistrian University of Athens, 2 Thivon Str, 11527 Athens, Greece; amitsea@dent.uoa.gr (A.M.); kkara@dent.uoa.gr (K.K.)

**Keywords:** dentin dysplasia type I, impacted canines, orthodontic treatment, genetic analysis, aesthetic restoration

## Abstract

Dentin dysplasia is a rare hereditary disorder, transmitted by autosomal dominant mode, affecting both dentin and pulp. In Type I crown morphology is normal, but root dentin organization loss leads to shorter roots. Mutations in the *SSUH2*, *VPS4B* and *SMOC2* genes have been reported as responsible for this condition. Orthodontic treatment was conducted on an 11-year-old female patient presenting the disorder along with bilaterally impacted permanent maxillary canines, in close proximity to the roots of the lateral and central incisors. Treatment plan included lateral incisors extraction, surgical exposure and traction of the impacted canines. Light forces were applied from a custom-made trans-palatal arch. Comprehensive orthodontic treatment was performed using edgewise appliances. After 3 years and 2 months, group function occlusion was achieved. The canines underwent composite resin restorations. At one year post-retention, the dentition remained stable. Family-based genetic analysis did not reveal any mutations in the aforementioned genes pointing to further genetic heterogeneity of this disorder. As dental medicine becomes more sophisticated and personalized, the association between mutation type/function and orthodontic treatment response may provide useful therapeutic insights. The positive treatment response of the presented case could be attributed to a more “benign” mutation awaiting to be identified.

## 1. Introduction

Dentin Dysplasia type I (DD-I) is a rare genetic developmental disorder that may exhibit an autosomal dominant pattern of inheritance and occurs in 1:100,000 individuals [1,2]. It affects both the primary and permanent dentitions [3,4,5].

Clinically, the teeth present nearly normal crown morphology. However, malformed, short or absent roots [6], tooth mobility [7] or premature exfoliation may be observed. Delayed tooth eruption has also been reported in some cases [6,7].

Radiographic findings are key diagnostic tools in identifying the condition [8]. Roots appear to be short, blunt, tapered or even absent [6,7]. Obliteration of pulp chambers is also observed, with crescent-shaped pulp remnants parallel to the cementoenamel junctions [8]. Roots with obliterated pulp canals, as well as periapical radiolucent areas, associated with absence of carious lesions, or any other kind of pathology, are common findings in patients with DD-I [4,6,7,8,9,10].

O’Carroll et al. [10] have introduced a subclassification of DD-I, based on radiographic characteristics: DD-Ia presents complete obliteration of the pulp, little or no root development, periapical lesions and tooth exfoliation. In DD-Ib, roots are extremely short and conical, presenting a horizontal crescent-shaped line along the cementoenamel junction. In DD-Ic, roots are half their normal length with two crescent-shaped horizontal lines concave towards each other. DD-Id roots have normal length and a pulp chamber is evident at the cementoenamel junction. Pulp stones are common findings.

The characteristic pattern of DD-I indicates that a genetic background is responsible for the condition. SPARC-related modular calcium binding protein 2 (SMOC2) is a protein encoded by the SMOC2 gene, which encompasses 14 exons, lies in chromosome 6q27 and has been shown to be mutated in similar dental developmental defects [11]. Vacuolar protein sorting 4 homolog B (VPS4B) gene is located in chromosome 18 and has been identified as a disease-causing gene for DD-I [12]. Furthermore, a study using a large Chinese family with 14 DD-I patients revealed a missense mutation in the Ssu-2 homolog (SSUH2) gene, that disrupts normal dental formation and causes autosomal dominant DD-I [13]. These articles suggest that different mutations can result in the same phenotype and that DD-I exhibits genetic heterogeneity [14]. Identification of specific genetic variants on DD-I relevant genes associated with successful treatment results could allow clinicians to conduct treatment that would otherwise be considered venturesome.

Although there are several dental case reports in the literature on patients with DD-I [5,6,15,16,17], there is little evidence of patients being treated with comprehensive orthodontic treatment [18]. The purpose of this article was to describe the orthodontic treatment of a female adolescent patient with DD-I, also presenting with two maxillary impacted canines. Clinical considerations about the treatment plan were discussed, as well as the limitations set by this disorder. Furthermore, the presence of the most common DD-I gene mutations was investigated.

## 2. Case Presentation

An 11-year-old girl with a chief complaint of non-erupted maxillary permanent canines was referred for treatment to the Orthodontic Clinic of the Dental School. Her father was diagnosed with Dentin Dysplasia Type I. Permission was obtained from father and daughter to use the diagnostic records (initial, progress, final), including photographs and X-rays, for scientific reasons.

### 2.1. Clinical Findings and Diagnosis

**Figure 1:**
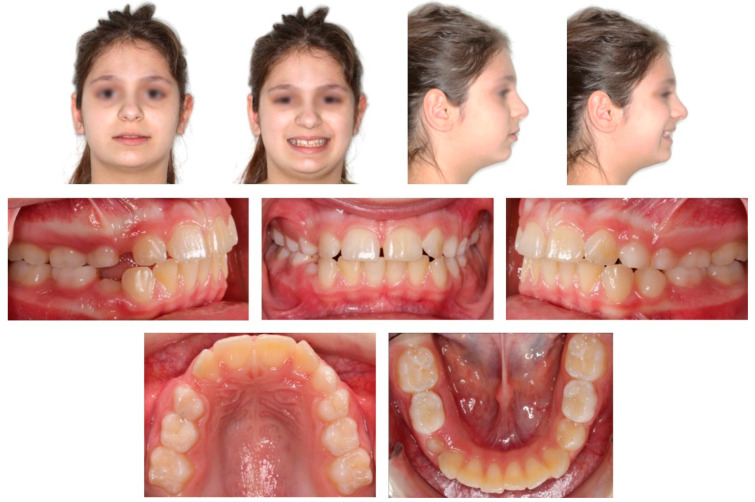
Pre-treatment extra- and intra-oral photographs.

Extra-oral examination of the child revealed a convex profile, slightly reduced lower facial height and increased labionasal angle. Intra-oral clinical examination showed a mixed dentition in Class II molar relationship, reduced overjet (2 mm) and overbite (10%) (Figure 1). Patient’s oral hygiene was good. 

**Figure 2:**
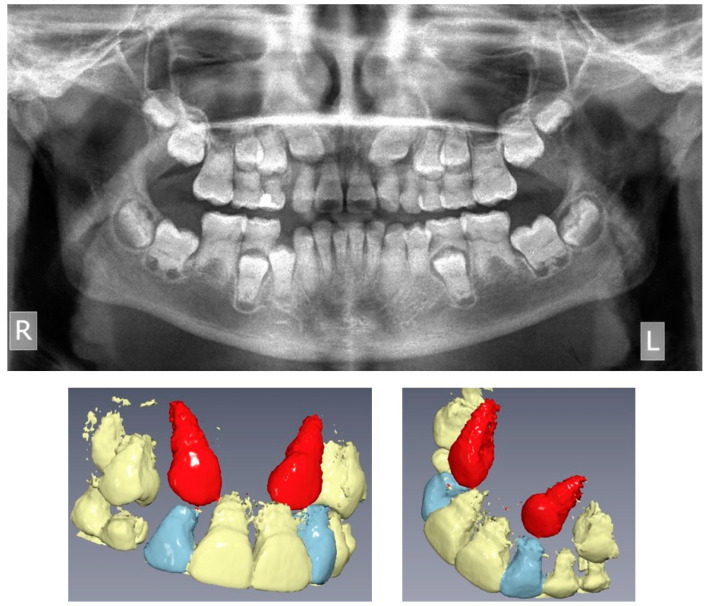
Pre-treatment panoramic radiograph and Cone Beam Computed Tomography 3D reconstruction of the maxilla, showing the impacted upper canines (R: right, L: left, upper canines displayed in red).

**Figure 3:**
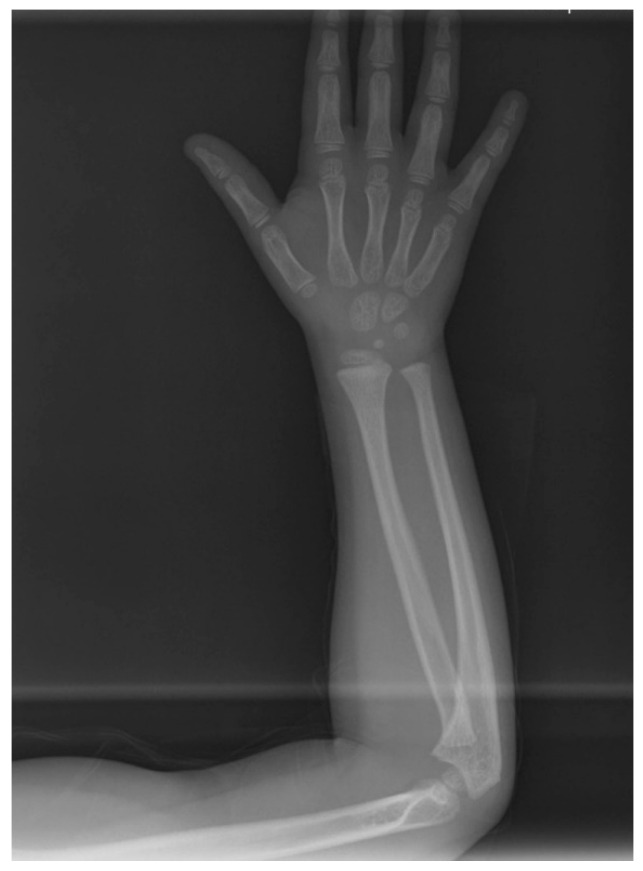
Hand-wrist and long bone radiographic presentation of the daughter exhibiting no osseous defects.

The pre-treatment panoramic radiograph revealed short root length of most of the teeth, delayed tooth eruption, obliteration of pulp chambers and periapical radio-lucencies, not associated with periodontal disease or pulp inflammation. Horizontal crescent-shaped lines along the cementoenamel junction were evident in several teeth. Based on radiographic characteristics, this patient was subclassified as DD-Ic [10]. The permanent maxillary canines were impacted (Figure 1). Pre-treatment cone-beam-computed-tomography scans showed that the upper right maxillary canine (#13) was labially impacted; the contralateral canine (#23) displayed a more palatal position. Both teeth were above the roots of the adjacent lateral incisors, in close proximity to them and to the roots of the central incisors (Figure 2). Sclerosis of the maxilla and the mandible was absent in this case, as well as in the skeleton (Figure 3).

**Figure 4:**
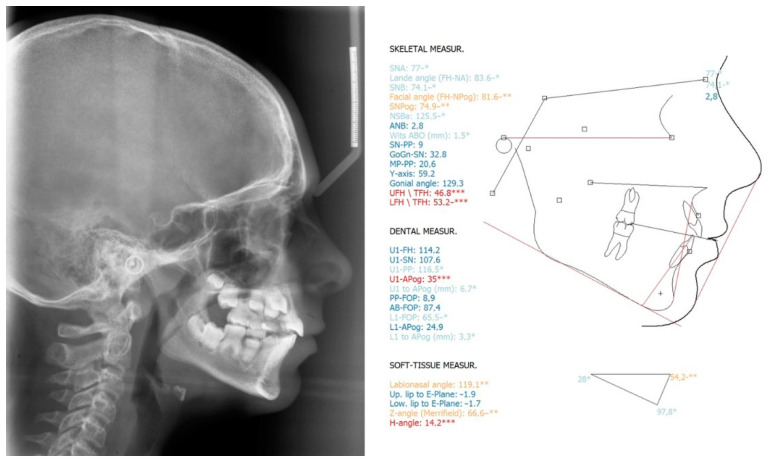
Pre-treatment lateral cephalometric radiograph and cephalometric analysis. *: ±1 Standard Deviation from the norm (light blue), **: ±2 Standard Deviations from the norm (orange), ***: ±3 Standard Deviations from the norm (red).

Pre-treatment cephalometric analysis showed no considerable deviations from the norms, except for the anterior facial height (UFH/TFH = 47%; LFH/TFH = 53%), facial convexity (Z-angle = 67°), inclination of the lower central incisors (IMPA = 98°) and labionasal angle (119°) (Figure 4).

### 2.2. Treatment Objectives

The diagnosis was set from clinical and pre-treatment radiographic findings. Treatment plan included extraction of the upper lateral incisors, surgical exposure of the impacted canines and canine substitution of the extracted lateral incisors. The upper lateral incisors were extracted, since they presented poor long-term prognosis, due to the short, malformed roots and the reduced crown to root ratio, not only because of the disorder, but possibly also because of the interference of the upper canines with the roots of the lateral incisors.

### 2.3. Treatment Planning

**Figure 5:**
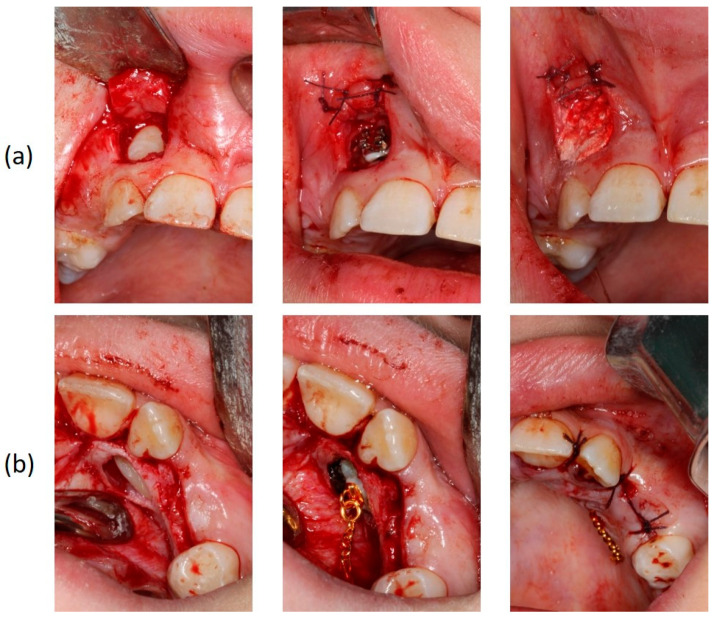
Surgical exposure of the upper right (**a**) and left impacted canines (**b**).

**Figure 6:**
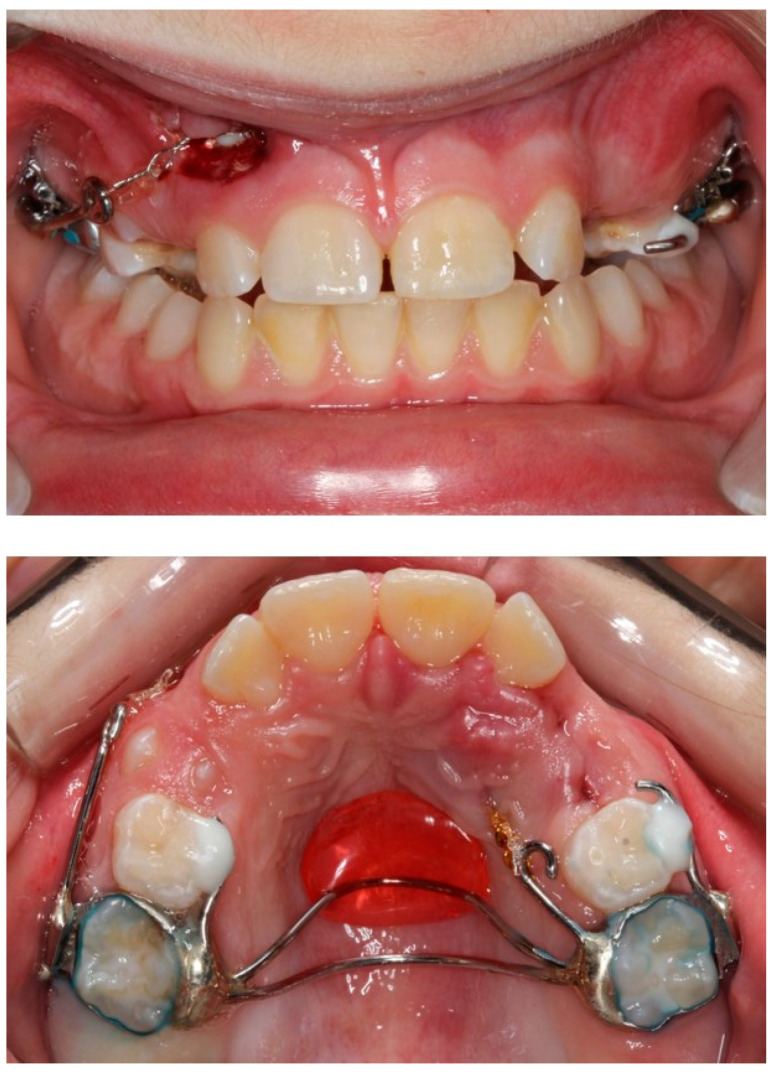
The custom-made trans-palatal arch used for the traction of the impacted canines.

**Figure 7:**
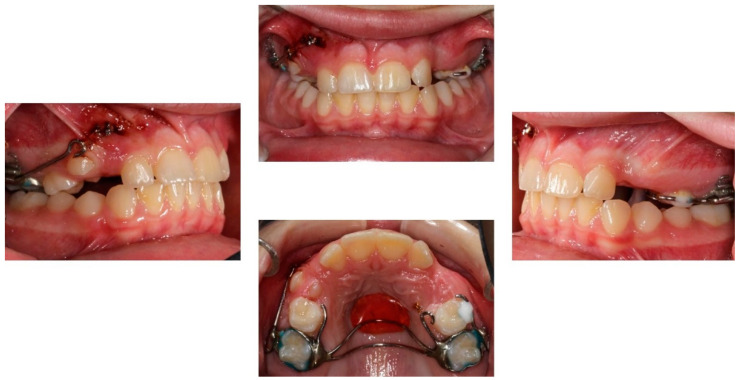
Progress photographs of the custom-made trans-palatal arch used for the traction of the impacted canines.

**Figure 8:**
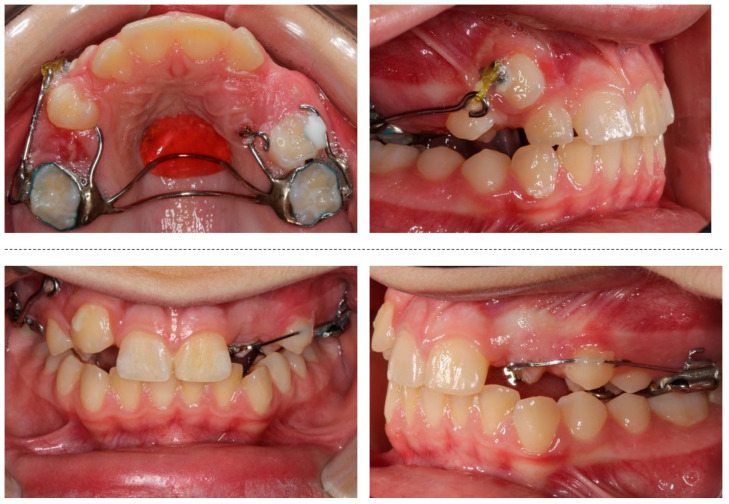
Sectional mechanics used for the traction of the impacted canines.

The orthodontic treatment was initiated with the canine exposure (Figure 5) and traction with light forces using a custom-made trans-palatal arch for anchorage purposes (Figure 6 and Figure 7). An open surgical technique (apically positioned flap) was used to expose the labially positioned right canine and a closed technique for the left, which was positioned palatally. Sectional mechanics were applied during treatment until the eruption of all permanent teeth (Figure 8). The upper lateral incisors were extracted after the successful eruption of the impacted canines and for aesthetic reasons. Subsequently, comprehensive orthodontic treatment was performed using fixed 0.018-inch-slot edgewise appliances. A lower lingual arch was placed to preserve the leeway space thus helping with the lower crowing. The progression of the archwire sequence was from 0.014-inch nickel-titanium to 0.016-inch Australian archwires. Class III light elastic forces were used bilaterally to help with space closure on the upper arch.

### 2.4. Treatment Results

**Figure 9:**
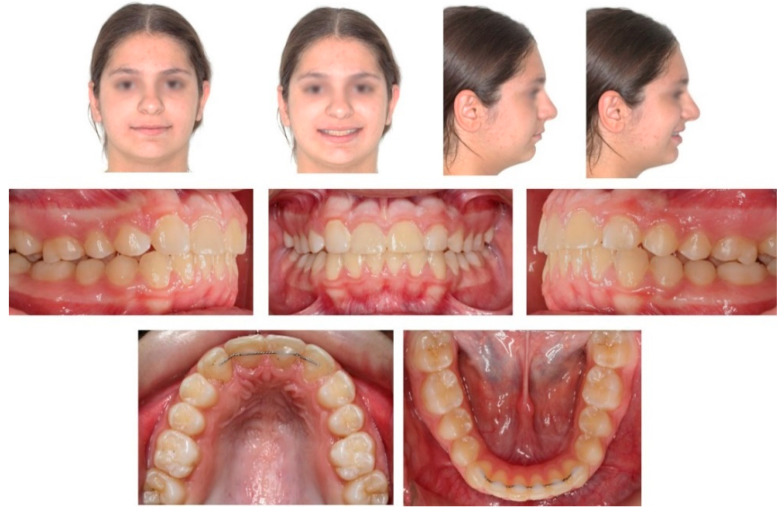
Post-treatment extra- and intra-oral photographs.

After a total treatment of 3 years and 2 months, a group function occlusion was achieved, with positive overjet and overbite. No interferences in the non-working side were present with lateral movements. The canines underwent composite resin restorations to resemble the lateral incisors (Figure 9).

**Figure 10:**
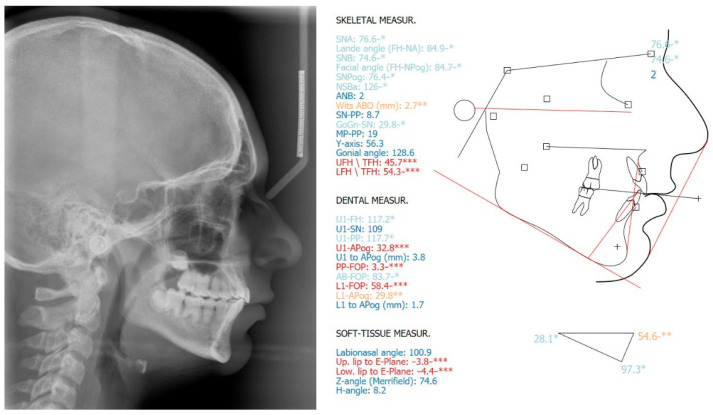
Post-treatment lateral cephalometric radiograph and cephalometric analysis. *: ±1 Standard Deviation from the norm (light blue), **: ±2 Standard Deviations from the norm (orange), ***: ±3 Standard Deviations (red).

The post-treatment lateral cephalometric analysis did not reveal considerable changes (Figure 10). Upper and lower anterior facial heights remained approximately the same (UFH/TFH = 46%; LFH/TFH = 54%), and so did the inclination of the lower incisors (IMPA = 97°). Labionasal (100°) and Z-angles (75°) improved and a more pleasant soft tissue profile was established.

**Figure 11:**
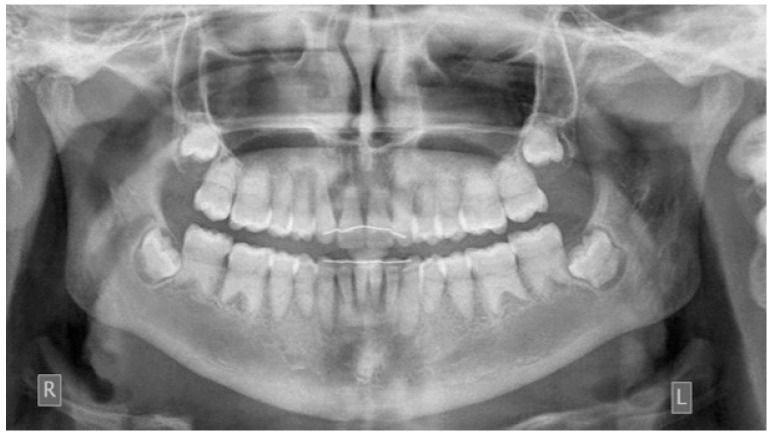
Post-treatment panoramic radiograph. No root resorption was observed (R: right, L: left).

The post-treatment radiographic evaluation showed no further root resorption (Figure 11).

### 2.5. Retention

**Figure 12:**
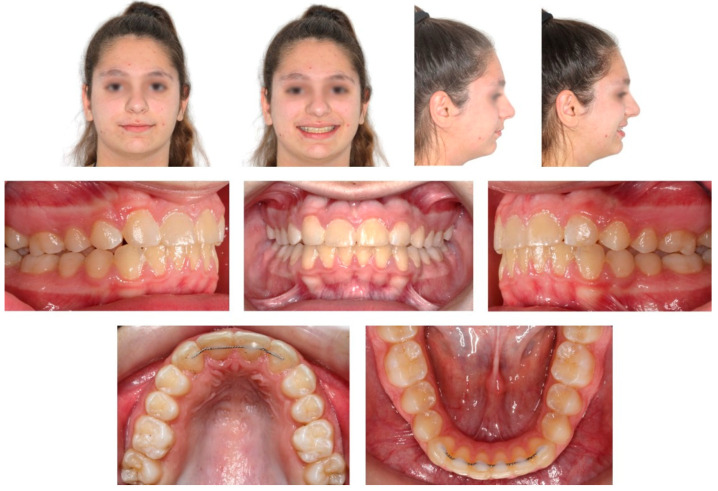
Extra- and intra-oral photographs at one-year post-debonding. Treatment results remained stable.

The retention protocol included bonded retainers between #13–23 and #33–43 (0.0215-inch twistflex), as well as Hawley and Essix appliances on the upper and lower arch, respectively. Treatment results remained stable at one-year post-retention (Figure 12).

## 3. Genetic Analysis of the Family

### 3.1. Saliva Sampling, DNA Isolation, PCR and Sanger Sequencing

The most common genetic mutations responsible for the DD-I are the c.84 + 1G > T mutation in the *SMOC2* gene [11], the IVS7 + 46C > G mutation in the *VPS4B* gene [12], and the c.353C > A mutation in the *SSUH2* gene [13]. Thus, we aimed to investigate the presence of these mutations. Family dental history revealed that the present patient (proband) shared the same phenotypic characteristics of the condition with the father, but not with the mother. The protocol was approved by the Ethics Committee, Dental School (No. 357/29 March 2018). Written informed consent was obtained from all three members of this family.

Saliva samples were collected from all three family members and DNA extraction was performed (QIAamp DNA Blood Mini Kit, Qiagen, Hilden, Germany), according to the manufacturer’s instructions. DNA concentration was determined using a NanoDrop One spectrophotometer (ThermoFisher Scientific, Waltham, MA, USA). Primers and PCR conditions used for amplifying intron 1 of the SMOC2 gene, the region between exon 7 and exon 8 of the VPS4B gene and the exon 2 of the SSUH2 gene were described elsewhere, respectively [11,12,13]. Both forward and reverse PCR products for all three genes were directly sequenced, using a 3730xl DNA analyzer (Applied Biosystems, ThermoFisher Scientific). All chromatograms produced from sequencing were examined.

### 3.2. Results of the Genetic Analysis

**Figure 13:**
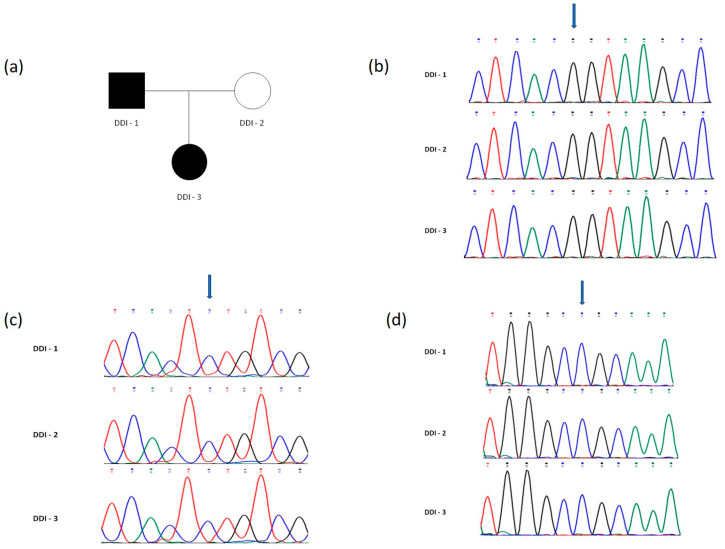
(**a**) Pedigree of the family. Affected subjects are denoted in black. DDI-3 is the proband. (**b**–**d**) DNA sequence chromatograms obtained from Sanger sequencing revealing normal status of (**b**) the c.84 + 1G > T mutation in the SMOC2 gene, (**c**) the IVS7 + 46C > G mutation in the VPS4B gene and (**d**) the c.353C > A mutation in the SSUH2 gene, in all three family members (DDI-1, DDI-2 and DDI-3). Mutation sites previously reported are indicated by blue arrows.

No mutations were found in any of the samples examined (Figure 13).

## 4. Discussion

### 4.1. Orthodontic Treatment

The orthodontic treatment in this patient was necessary due to the extremely unfavorable position of the upper canines. Many case reports of impacted upper canines in the orthodontic literature demonstrated root resorption of the central and lateral incisors [19,20,21,22,23]. Resorption of the central incisors would be devastating in this case, due to the poor long-term prognosis of the entire dentition.

The dental age and the adverse tooth prognosis in this patient necessitated the use of a custom made trans-palatal arch for the traction of the canines. This arch was bonded on the permanent first molars, as well as the palatal and buccal surfaces of the right and left deciduous second molars, respectively. The appliance was further stabilized on the hard palate mucosa with an acrylic button. This design allowed the even distribution of the reciprocal forces during the canine traction on the aforementioned structures.

Orthodontic treatment is challenging in patients with DD-I, mainly because of the potential adverse effects of the forces on the short, malformed roots. Comprehensive orthodontic treatment and heavy forces may induce root resorption [24,25]. The heavier the forces applied, the larger the total root resorption volume, as shown by microCT imaging techniques [26]. The treatment plan and the biomechanics selected for this patient aimed to avoid root resorption: entity of tooth forces was kept to low levels, a custom made trans-palatal arch was used for the traction of the canines and only round archwires/light forces were applied. The present patient exhibited no caries or tooth mobility, maintained vital pulp response and a healthy periodontium. The upper right canine showed a minor recession after alignment, although the height of the attached gingiva during surgical exposure was preserved with an apically positioned flap. Further extrusion of this tooth was excluded due to the root morphology. Alternative treatment plans including upper distalization or extractions in the lower arch were not considered due to the poor long-term prognosis of the entire dentition.

Under these conditions and for functional reasons, full orthodontic treatment could be justified in patients with DD-I, providing all risks are meticulously evaluated and thoroughly explained to the patient and the family before the onset of treatment, and carefully monitored during the course of treatment.

### 4.2. Differential Diagnosis

In the present case, the diagnosis was based on the clinical and radiographic findings, as well as on the patient’s and family dental history. Dentin defects are classified into Dentinogenesis Imperfectas (DIs, types I–III) and Dentin Dysplasias (DDs, types I, II) [27,28]. Radiographically, it resembles the phenotype of DD-I; narrow roots and small or obliterated pulp chambers and root canals are common findings. Teeth appear blue-gray or amber brown and opalescent, characteristics that were not evident in our case. DI type I is also accompanied by osteogenesis imperfecta, a disorder of bone fragility, and DI type II by hearing loss [27,28]. The present patient exhibited neither bone/hearing defects according to her medical history nor any of the clinical dental characteristics of DI. Therefore, this entity was excluded from the differential diagnosis.

The genetic analysis of the family members did not reveal any of the most common mutations for DD-I. All family members showed identical sequence pattern. Absence of known mutations indicates that novel mutations in the same genes or other encoding products for dentin formation may be responsible for the phenotypic variability of DD-I. An extended pedigree was not acquired for this patient because of the few family members and lack of dental history for the previous generations.

Other genes have also been reported to be involved in dentin formation. *Dentin sialophosphoprotein* gene (*DSPP*) encodes three major non-collagenous dentin matrix proteins: the dentin sialoprotein (DSP), the dentin glycoprotein (DGP) and the dentin phosphoprotein (DPP) [27]. *DSPP* is expressed a hundred-fold more in dentin than in other tissues [27]. Mutations in *DSPP* have been found to be the major cause for DI types II and III and DD type II [27,29,30,31]. Normal teeth crown morphology excluded this gene from being a proper candidate to examine.

Type I collagen forms the organic phase of the dentin [27]. Most cases of osteogenesis imperfecta are associated with mutations in the procollagen type I genes *COL1A1* and *COL1A2*, leading to abnormal collagen I fibrils formation [32]. However, the clinical characteristics of osteogenesis imperfecta do not resemble these of the present case.

Enamel malformations include a wide clinical spectrum of enamel phenotypes; hypoplastic, hypocalcified, hypo-maturation, and hypo-maturation-hypoplastic with taurodontism, caused by known gene defects [33]. During tooth formation two proteinases are secreted by ameloblasts: *enamelysin* (*MMP-20*) and *kallikrein-4* (*KLK4*) [33]. Mutations of *MMP-20* and *KLK4* cause autosomal-recessive amelogenesis imperfecta (ARAI) [33]. Also, mutation in *FAM20A* gene resulted in hypoplastic amelogenesis imperfecta. Family with sequence similarity 20 (*FAM20*) includes three members (*FAM20A*, *FAM20B* and *FAM20C*). *FAM20A* encodes a secreted glycoprotein [34]. The most severe type, the hypocalcified amelogenesis imperfecta, is mostly caused by nonsense mutations in the *FAM83H* gene [35]. Nonetheless, the clinically normal crown morphology in this patient set enamel malformations very low to the differential diagnosis list.

### 4.3. Genetic Analysis

Genetic analyses in this family did not reveal any mutations in the *SSUH2*, *VPS4B* or *SMOC2* genes. Most reported DD-I cases are neither genetically studied nor treated orthodontically and, therefore, the mutation type cannot be associated with the orthodontic result. Reporting cases with genotype-phenotype associations could provide evidence on where good response to orthodontics is expected.

## 5. Conclusions

Orthodontic treatment can be cautiously conducted in patients with DD-I, when functional and other reasons pertaining to tooth survival demand it. In these cases, the clinical and radiographic examination should be carefully co-evaluated with the familial dental history. The patient’s chief complaint and the biomechanical limitations must be carefully considered. In the present case, the primary goal to maintain the anterior dentition was accomplished. Additionally, a familial genetic analysis was performed in order to enrich our understanding of the defective dental genes and eventually contribute to a more personalized treatment. This combination concept of a case report/genetic analysis is becoming more feasible and more essential in managing atypical orthodontic patients.

## Data Availability

Not applicable.
